# Population Genetic Investigation of *Hypophthalmichthys nobilis* in the Yangtze River Basin Based on RAD Sequencing Data

**DOI:** 10.3390/biology13100837

**Published:** 2024-10-18

**Authors:** Weitao Li, Jiongying Yu, Yanfu Que, Xingkun Hu, Ezhou Wang, Xiaolin Liao, Bin Zhu

**Affiliations:** 1 Key Laboratory of Ecological Impacts of Hydraulic-Projects and Restoration of Aquatic Ecosystem of Ministry of Water Resources, Wuhan 430079, China; liweitao10@163.com (W.L.); yfque@mail.ihe.ac.cn (Y.Q.); huxingkun@mail.ihe.ac.cn (X.H.); wezsstsyy@mail.ihe.ac.cn (E.W.); 2 Institute of Hydroecology, Ministry of Water Resources and Chinese Academy of Sciences, Wuhan 430079, China; 3 School of Life Sciences, Huzhou University, Huzhou 313000, China; yujiongying@outlook.com; 4 Fisheries College, Hunan Agricultural University, Changsha 410128, China

**Keywords:** *Hypophthalmichthys nobilis*, RAD-seq, genetic diversity, population structure

## Abstract

The Bighead carp (*Hypophthalmichthys nobilis*), a primary freshwater aquaculture species in China, faces challenges due to over-exploitation and environmental changes. We leveraged RAD-seq to perform a comprehensive population genetic analysis on 14 *H. nobilis* populations sampled from the Yangtze River (13 populations) and the Marseilles Reach of the Illinois River (one population). Analysis of genetic diversity showed that different parameters demonstrated varied inferences, and notably, Zhongxian (ZX2), Wanhzou (WZ2), Yangzhou hatchery (YZYZ), Yangzhou (YZ), and Taihu (TH) populations showed apparent heterozygote deficiency. Linkage disequilibrium (LD) analysis exhibited a trend of higher linkage disequilibrium in populations from the upper reaches of the Yangtze River, followed by those from the middle reaches and then those from the lower reaches. Additionally, the reconstructed polygenetic tree and PCA plot clustered all populations into 2 major subgroups, while the results of structure analysis indicated 4 ancestors. The pairwise *F_ST_* values ranged from 0 to 0.5530. Among these, high *F_ST_* values (0.1931–0.5530) were only observed between populations WZ2, YZ, YZYZ, and the remaining 11 populations. Furthermore, genetic bottlenecks were observed in all populations 20–30 thousand years ago. Overall, the research offers insights essential for genetic management practices for sustainable aquaculture and biodiversity conservation of bighead carp.

## Simple Summary

The Bighead carp (*Hypophthalmichthys nobilis*) is an economically important freshwater species in China. To promote scientific management strategies and guide the future monitoring of *H. nobilis* genetic resources in the Yangtze River, we genotyped multiple bighead carp populations sampled alongside the Yangtze River in China using RAD sequencing, explored their genetic diversity and population structure, and investigated their population demographic history. The findings in the study will make a meaningful contribution to the conservation and utilization of bighead carp germplasm resources in China.

## 1. Introduction

Bighead carp (*Hypophthalmichthys nobilis*), taxonomically classified in Cypriniformes, Cyprinidae, and *Aristichthys*, is a highly valued freshwater aquaculture species in China, ranking third in fish production with an annual yield of 3.27 million tons in 2022 [1]. As a semi-migratory species, growing in floodplain lakes and spawning in large rivers, *H. nobilis* is natively distributed in the Yangtze River, Qiantang River, Pearl River, Yellow River, and Haihe basin in China and has been introduced to over 70 countries since the 1970s due to its economic and ecological significance [2,3]. Among them, the Yangtze River, being the largest and longest river in China, has historically been the primary habitat for *H. nobilis*. Prior to the advent of artificial propagation in 1958, juvenile fish used for aquaculture were predominantly sourced from the Yangtze River. Presently serving as a vital germplasm resource bank of *H. nobilis*, the Yangtze River supplies high-quality broodstock to those national hatcheries located near the middle and lower reaches [4]. Unfortunately, over-exploitation and water pollution have precipitated a significant aquatic biodiversity crisis in the Yangtze River. Additionally, hydrological changes induced by hydraulic projects, such as alterations in temperature, water chemistry, and water levels, have adversely affected the migration and reproduction of *H. nobilis* and hindered its genetic exchange across different watersheds [5,6].

Continuous efforts have been made in recent years to conserve water bio-resources and recover biodiversity. Among those initiatives, restocking and stock enhancement through releasing hatchery-reared juveniles into the wild has been considered a tantalizing tactic in response to the depletion of germplasm resources, which has been applied to species such as *Nibea japonica* [7], *Penaeus penicillatus* [8] in China, *Garra cambodgiensis* [9] in Thailand, *Paracentrotus lividus* [10] in Italy. To restore the declining resources of *H. nobilis* and strike a balance between its conservation and exploitation, restocking and stock enhancement projects alongside the Yangtze River have been ongoing since the 1950s [11]. However, the absence of scientific management results in the release of fingerlings from unidentified sources during the stocking process, inevitably affecting the genetic structure of wild populations. Moreover, some hatcheries encounter issues, including small breeding populations and significant inbreeding during artificial reproduction, which adversely affect the quality of fingerlings and degrade the economic traits of *H. nobilis* [12].

To design effective breeding programs and provide scientific guidance for restocking and stock enhancement, a thorough investigation into the genetic background of *H. nobilis* populations is critical. The limited studies carried out thus far usually focused on genetic evaluation using morphological characteristics [3], isoenzymes [13], mitochondrial DNA [14], RELP [15], or microsatellites [12,16,17]. Nonetheless, a comprehensive understanding of the genetic structure of *H. nobilis* populations along the Yangtze River remains elusive due in part to limited molecular markers and small sample sizes in the previous literature. Notably, the advent of high-throughput sequencing technologies is revolutionizing the field of genetics by providing organism’s detailed genotyping data. Among them, the RAD-seq (Restriction-site associated DNA-sequencing) technique, involving digesting genomic DNA with restriction enzymes followed by sequencing the digested fragments, is characterized by its simple workflow, low cost, and independence from the reference genome and has been widely applied to the genetic map construction [18,19], genetic dissection of economic traits [20,21] and population genetic studies [22,23,24].

In this study, we utilized RAD-seq to perform the population genetic analysis of *H. nobilis* in the Yangtze River. Using genome-wide DNA markers, we investigated their genetic diversity, evaluated the population differentiation, and provided genomic evidence on the historical population dynamics, which are vital for formulating scientific management strategies and guiding the future monitoring of *H. nobilis* genetic resources in the Yangtze River.

## 2. Materials and Methods

### 2.1. Sampling

**Figure 1:**
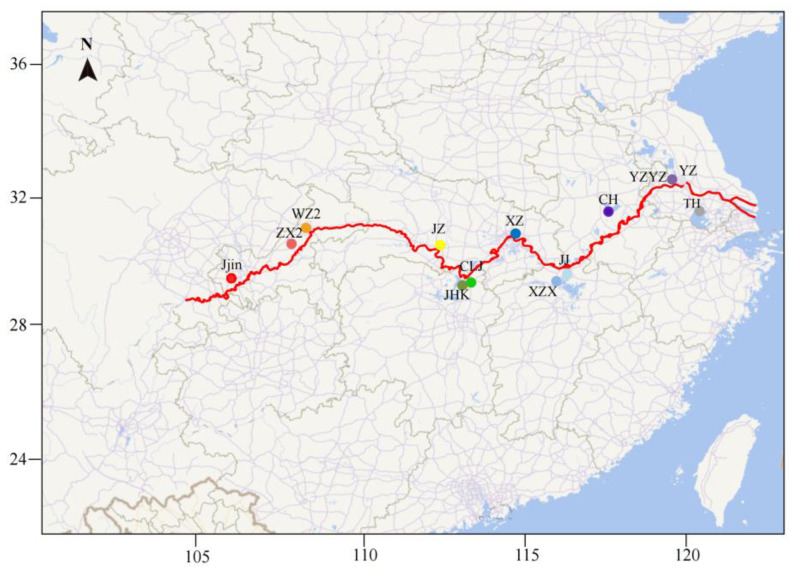
Geographic map showing the sample sites (solid circles) along the Yangtze River. YZYZ was sampled from the broodstock of a national hatchery, and the remaining populations were sampled from the wild.

The Yangtze River Basin, spanning a total length of 6300 km, is divided into three reaches—upper, middle, and lower—based on geographical environment and hydrological characteristics. From upper to lower reaches, the current slows to a gentle flow while human activity steadily picks up. Considering the ecological characteristics of the Yangtze River basin and the distribution patterns of bighead carp, 13 geographic populations along the Yangtze River and one population in the Marseilles Reach of the Illinois River were sampled in 2017. At each sampling site, *H. nobilis* individuals weighing 1 to 2 kg were sampled using fishing nets. The individuals were identified by their typical and unambiguous morphological characteristics, including the big head with a length exceeding 30% of the total length and the abdominal keel extending from the base of the ventral fin to the anus. Following the collection of tail fin clips from each individual, preserved in 95% ethanol and stored at −20 °C, the individuals were subsequently released back into their environment. Collectively, a total of 157 individuals were sampled. Detailed sampling information is presented in Table 1 and Figure 1.

### 2.2. RAD-Seq Library Preparation and Sequencing

The genomic DNA of each specimen was extracted using a DNA extraction kit (Tiangen, Beijing, China) according to the manufacturer’s instructions. The integrity and purity of DNA were assessed using 0.8% agarose electrophoresis and NanoDrop 2000 spectrophotometer (Thermo Fisher Scientific, Waltham, MA, USA), respectively, and the DNA concentration was determined using a Qubit 2.0 fluorometer (Life Technologies, Gaithersburg, MD, USA). High-quality DNA was used to construct the RAD-seq library at Wuhan Frasergen Bioinformatics Co., Ltd. (Wuhan, China). Briefly, 100 to 1000 ng of genomic DNA from each specimen was digested with the EcoRI restriction enzyme, and the digested fragments were ligated to the Solexa P1 adapter. The samples were then pooled together and randomly sheared ultrasonically, and fragments ranging from 300 bp to 700 bp were selected using agarose gel electrophoresis. Following the ligation to the Solexa P2 adapter, additional PCR amplification was performed to enrich fragments containing adapters at both ends. After pooling according to the effective concentration and target data volume, the qualified libraries were sequenced on an Illumina HiSeq2000 system (Illumina Inc., San Diego, CA, USA) with 150 bp paired-end reads.

### 2.3. Genotyping

To ensure high-quality data for subsequent analysis, the obtained raw data were filtered using the fastp program [25]: trimming bases with a Phred quality score of less than 20 and removing reads with adapters or with lengths less than 50 bases. Then, the obtained clean data were aligned to the reference genome of *H. nobilis* (HypMol1.0 with a Genebank number of GCA_004193235.1) using BWM-MEM (v 0.7.17) with default parameters [26]. The alignment results were sorted using Samtools (v1.9), and PCR duplicates were removed using MarkDuplicates in Picard Tools (v2.13.2) (http://broadinstitute.github.io/picard/) (accessed on 17 September 2024). SNP calling for each individual was then carried out using GATK HaplotypeCaller (v4.1.4.1) [27] with default settings. To ensure the reliability of the subsequent analysis, we filtered the calls using GATK VariantFiltration with the following parameters: QD < 2.0, FS > 60.0, MQ < 40.0, MQRankSum < −12.5, ReadPosRankSum < −8.0. The SNP calls for each sample were then combined using GATK CombineGVCFs under default settings. Each SNP was further assessed and filtered at the population level if (1) minor allele frequency < 0.01, (2) samples with missing genotypes > 0.2, and (3) sequencing depth < 4. The remaining SNPs were used to perform the population genetic analyses.

### 2.4. Genetic Diversity and Linkage Disequilibrium Analysis

Genetic parameters were analyzed using VCFtools (v0.1.13) [28], including nucleotide diversity (*Pi*), observed heterozygosity (*HO*), expected heterozygosity (*HE*), and the inbreeding coefficient (FIS). *Pi* was analyzed using a sliding-window approach with a window size of 5 kb. The Hardy–Weinberg equilibrium (HWE) test for each population was also performed using VCFtools. Additionally, PopLDdecay (v3.40) was used to perform linkage disequilibrium (LD) analysis based on the filtered SNP data [29].

### 2.5. Population Differentiation and Structure Analyses

To determine genetic differentiation among all sampled populations, the pairwise F-statistics (*F_ST_*) among populations were calculated, and nonparametric multivariate analysis of variance (non-parametric MANOVA) was performed using the function adonis in the package Vegan [30]. To further enhance comprehension of the sampled *H. nobilis* population, we utilized Admixture v1.3.0 software [31] to analyze the population structure, in which the putative number of genetic groups (K-value) was assumed to be 1–8 and ten independent runs for each K-value were conducted. The K-value with the minimum cross-validation error (CV error) was chosen as the best population structure. A phylogenetic tree was constructed using the neighbor-joining (NJ) method with Treebest software 1.9.2 [32]. Bootstrap analysis, repeated 1000 times, was employed to assess the reliability of the NJ tree, and the phylogenetic tree was plotted using iTOL (https://itol.embl.de) (accessed on 17 September 2024). Principal components analysis (PCA) was then performed using GCTA (1.91.4) [33], and the results were plotted with the first and second eigenvalues using the “ggplot2” package in R [34]. The Markovian Coalescent (MSMC) model [35] was used to estimate effective population size using heterozygous sites across the genome.

## 3. Results

### 3.1. Sequencing and SNP Calling

A total of 221 Gb clean bases with an average Q20 of 97.0% and an average Q30 of 91.7% were obtained (Table S1). Alignment to the reference genome depicted an average sequencing depth of 14.6× and an average sequencing coverage of 15.8%. Following SNP detection and high-quality filtering, a total of 1,405,783 SNP loci were retained for further analysis. All SNPs were categorized as either transitions (Ti) or transversions (Tv), with transversions accounting for 54.7% of the SNP sites and an observed Ti: Tv ratio of 1.235.

### 3.2. Results of Genetic Diversity and LD Analysis

Important genetic parameters were calculated to assess the genetic diversity using the high-quality SNPs (Table 2). The results revealed that *Pi* ranged from 0.0002 (BH, CLJ, JJ, Jjin) to 0.0013 (YZ and YZYZ), which demonstrated relatively low genetic variation at the nucleotide level across all populations. The expected heterozygosity (*HE*) varied from 0.2116 to 0.3997, with the highest value in XZX and the lowest value in ZX2. The observed heterozygosity (*HO*) was lowest in CH (0.0656) and highest in YZ (0.4248). The expected heterozygosity of loci was greatly reduced relative to the observed heterozygosity in populations ZX2, WZ2, YZ, YZYZ, and TH. Additionally, the number of loci showing significant deviations from the Hardy-Weinberg equilibrium among the 14 populations ranged from 4663 (JZ) to 919,153 (YZ). The inbreeding coefficient (*F_IS_*), occupied in determining the extent of inbreeding within a population, depicts the lowest value in JJ (−0.2905) and the highest value in YZ (0.8131).

**Figure 2:**
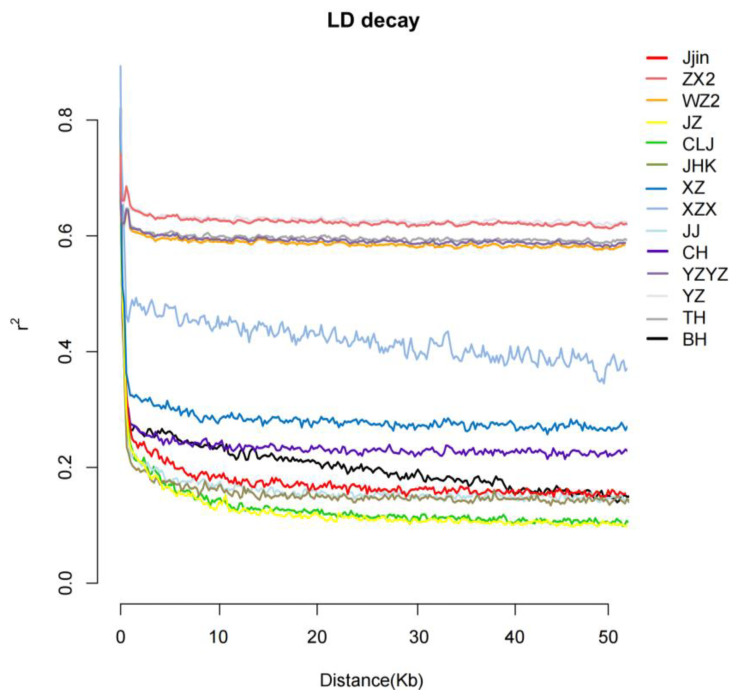
Patterns of linkage disequilibrium (LD) decay.

Analysis of LD decay revealed that all populations generally exhibited three levels of LD decay rate. Among them, the American population (BH) and six populations of the Yangtze River (Jjin, WZ2, JZ, CLJ, JHK, CH) displayed the fastest LD decay, with r^2^ = 0.5 at distances less than 10 kb. XZ and XZX exhibited moderate decay rates, also reaching r^2^ = 0.5 at distances less than 10 kb. While populations ZX2, JJ, YZ, TH, and YZYZ displayed the slowest decay rate, and the r^2^ value gradually stabilized around 0.6 as the physical distance between markers increased. The LD decay rate generally exhibited a trend of being higher in populations from the upper reaches of the Yangtze River, followed by those from the middle reaches and then those from the lower reaches (Figure 2).

### 3.3. Results of Genetic Differentiation and Population Structure

**Figure 3:**
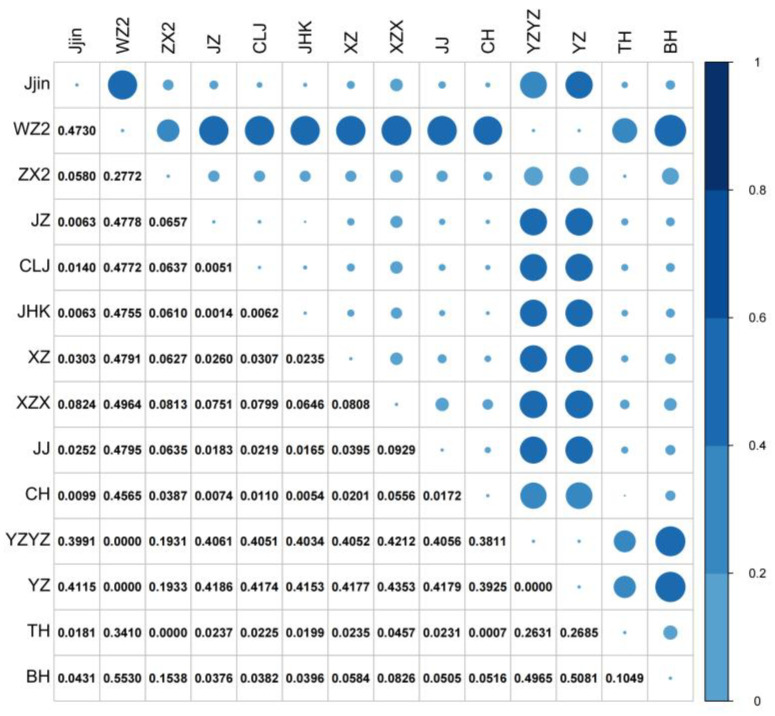
Pairwise *Fst* among the 14 sampled *H. nobilis* populations. The size of the dots is proportional to the represented *Fst* values.

The pairwise F-statistics (*F_ST_*) were calculated to investigate genetic differentiation. The pairwise *F_ST_* values among all populations ranged from 0 to 0.5530. High *F_ST_* values (0.1931–0.5530) were observed only between populations WZ2, YZ, YZYZ, and the remaining 11 populations. Low to moderate *F_ST_* values (0–0.1538) were observed within the three aforementioned populations and the remaining 11 populations (Figure 3). Additionally, results of nonparametric MANOVA showed 40.22% variation within individuals and 59.78% among populations (Table 3).

**Figure 4:**
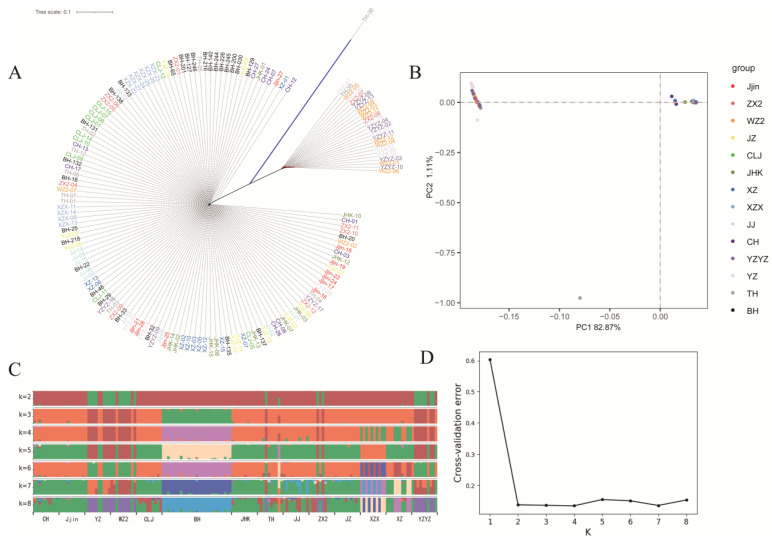
Results of population structure analyses. (**A**) phylogenetic tree for the 157 *H. nobilis* individuals. (**B**) PCA plot with the first and second eigenvector. (**C**) Result of the admixture analysis. The length of each colored segment represents the proportion of the individual genome inferred from ancestral populations (K = 2~8). (**D**) Cross-validation (CV) error for different K values in admixture analysis.

To further explore the genetic relationship among the investigated populations, population structure was analyzed. First, the reconstructed phylogenetic tree clustered the 157 *H. nobilis* specimens into two main clades. Clade 1 featured 25 individuals, of which 22 belonged to populations YZ, YZYZ and WZ2. Clade 2 featured 131 individuals, with individuals from the same geographical populations distributed across different branches (Figure 4A). Subsequently, the PCA plot, based on the first and second principal components explaining 82.87% and 1.11% of the total variance, respectively, revealed similar patterns of clustering (Figure 4B). Additionally, Admixture analysis indicated that the inferred genetic clusters of 2–4 were all presented with high probabilities. Nevertheless, all sampled populations were speculated to be oriented from four ancestral populations, which was supported by the cross-validation (when k = 4, the cross-validation was minimum). Specifically, populations YZ, YZYZ and WZ2 were dominated by the sample ancestor; population BH was dominated by an ancestor alone; partial specimens of populations YZYZ and ZX2 were dominated by the same ancestor; the remaining specimens were dominated by another common ancestor (Figure 4C,D).

### 3.4. Population Demographic History

**Figure 5:**
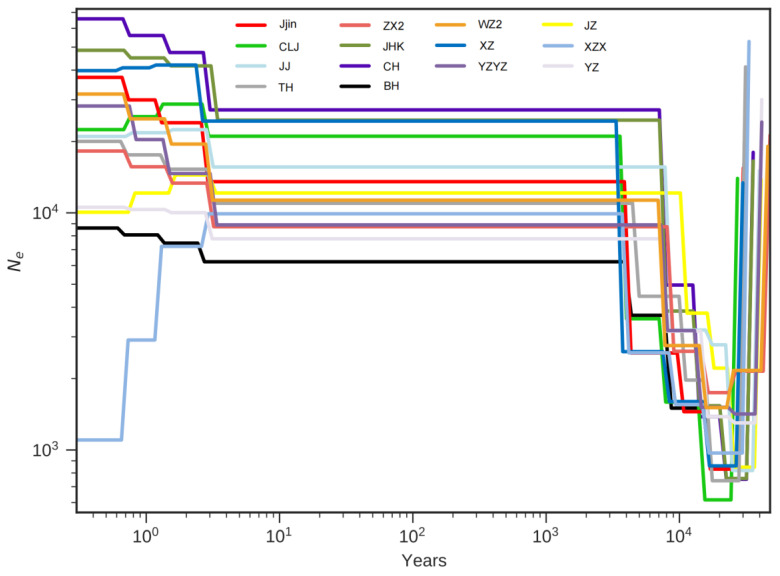
The estimated effective population size over time of the 14 *H. nobilis* populations using the MSMC model with a generation time of five years and a mutation rate of 2 × 10^−9^.

The MSMC model, with a generation time (g) of five years and a mutation rate per generation (μ) of 2 × 10^−9^, was used to estimate the historical effective population sizes (*Ne*). The reconstructed demographic history showed evidence that all populations experienced their lowest *Ne* between 20–30 thousand years ago, followed by a recovery to effective population sizes ranging from 6000 to 20,000 individuals. Subsequently, a remarkable stability in *Ne* was observed in all populations until a slight change occurred in the last 10 years (Figure 5).

## 4. Discussion

With the advancement of high-throughput sequencing techniques, research in fish population genetics has been increasingly important and popular in ecological, evolutionary, and conservation fields [24,36]. In the present study, we sampled 13 *H. nobilis* geographical populations from the Yangtze River basin and one American population, totaling 157 individuals, and performed population genetic analyses based on the 909,301 high-quality SNP loci obtained through the RAD-seq technique, dissecting the genetic background of *H. nobilis* in the Yangtze River at the genome-wide level.

Genetic diversity, reflecting the extent of genetic variation within a population and influenced by mutation, gene flow, genetic drift, or selection [37], has been considered essential for ensuring the evolutionary potential and environmental adaptability of a species [38,39]. Thus, genetic parameters, including nucleotide diversity, heterozygosity, and inbreeding coefficient, were initially calculated to determine the genetic diversity of the sampled populations. Expected and observed heterozygosity ranging from 0.2116 to 0.3997 and from 0.0656 to 0.4248, respectively, were observed in the 14 investigated populations, which were substantially lower than those reported in the previous literature using microsatellites [12,16,17,40]. A similar phenomenon was also observed in other fish species in the Yangtze River, such as *Mylopharyngodon piceus* [41], *Ctenopharyngodon idella* [42], *Misgurnus anguillicaudatus* [43], likely due to the use of different DNA markers. Furthermore, the nucleotide diversity analyzed in our study ranged from 0.2 × 10^−3^ to 1.3 × 10^−3^, falling into a similar range to that observed in *Megalobrama* (based on genome-wide SNP) [44] and grass carp (*Ctenopharyngodon Idella*) (based on mitochondrial DNA) [45], appearing to be very low overall, which might be due to the low mutation rate and long generation time (4–5 years in the wild) [46]. Nevertheless, the comparable expected heterozygosity to other *Cyprinidae* species that still maintain high genetic diversity, such as *Mylopharyngodon piceus* (*HE* = 0.273–0.292) [41], *Ctenopharyngodon Idella* (*HE* = 0.2523–0.2418) [42] and *Gymnocypris przewalskii* (*HE* = 0.3367–0.3444) [47], suggested that the *H. nobilis* populations investigated in our study still had high genetic diversity.

Among the 14 populations, populations ZX2, WZ2, YZ, YZYZ, and TH showed apparent heterozygote deficiency, with *HE* ranging from 0.2439 to 0.3997 and *HO* ranging from 0.0656 to 0.0897. such a large discrepancy was also observed in a mito-gynogenetic population of olive flounder (*Paralichthys olivaceus*) that was theoretically homozygous at all loci [48], indicating that these five populations might suffer strong inbreeding or the Wahlund effect [49,50,51], which was further confirmed by the high *F_IS_* value. This was the first time that such a high level of inbreeding coefficients has been documented in wild or farmed *H. nobilis* populations, which might be attributed to the high resolution provided by the genome-wide SNP markers. Usually, different from the heterozygote deficiency caused by null allele, the Wahlund effect or inbreeding should theoretically affect all loci equally, which was indeed observed in the five populations, wherein over 16% of loci in TH and over 50% of loci in YZ, YZYZ, WZ2, and ZX2 deviated from Hardy-Weinberg equilibrium. It was noteworthy that the aforementioned five populations exhibited higher expected heterozygosity and nucleotide diversity than the remaining populations despite showing higher inbreeding coefficients. Bjornerfeldt et al. [52] also reported such a rare phenomenon in a population of poodles and attributed the reason to the inclusion of multiple genetically distinct subgroups in the experimental population.

Linkage disequilibrium (LD) describes the non-random association of alleles at different SNPs within a given population and is extensively used to investigate the evolution and demographic process [53]. Typically, the rate of LD decay in a population is positively correlated with its genetic diversity, as observed in studies of *Megalobrama* [44], *Litopenaeus vannamei* [54], *Hyriopsis cumingii* [55] and *Pinctada fucata* [56]. However, the opposite was observed in the present study, wherein populations ZX2, WZ2, YZ, YZYZ, and TH, with higher nucleotide diversity and expected heterozygosity, exhibited the slowest LD decay rate, and the remaining populations also exhibited some degree of long-range LD. Similar phenomena were also reported in *Salmo salar* [57] and *Litopenaeus vannamei* [58], where admixture was suggested to be the major factor. The commonly recognized factors such as founder effect, inbreeding, selection, admixture, genetic drift, recombination as well as mutation are all key elements determining LD, thus the use of LD analysis alone to infer the genetic background of populations may generate spurious results. Preliminarily, based on the results of genetic diversity and LD Analysis, we observed a decline in genetic variation in ZX2, WZ2, YZ, YZYZ, and TH and speculated that YZYZ, sampled from the broodstocks in a national hatchery might have possessed admixed origin and suffered strong inbreeding, and ZX2, WZ2, YZ, TH were highly likely to have been undergoing events of admixture caused by large-scale restocking and stock enhancement activities.

To better understand the genetic structure of *H. nobilis*, pairwise *Fst* values were calculated, and analysis of the phylogenetic tree, STRUCTURE, and PCA were performed. Significant genetic differentiation, with high *F_ST_* values ranging from 0.1931 to 0.5530, was detected between populations YZYZ, YZ, WZ2 (assigned as group A) and the remaining 11 populations (assigned as group B), while low genetic differentiation was detected within the two groups, respectively. The reconstructed phylogenetic tree and PCA plot also demonstrated similar results, with YZYZ, YZ, and WZ2 clustering into a single subgroup and the remaining populations clustering into another subgroup. Additionally, the result of structure analysis further divided the 14 population into four ancestral sources, wherein the BH population was dominated by an ancestor alone, in agreement with the previous report [59], suggesting that prolonged geographical isolation had led to a certain degree of genetic differentiation. Notably, in the PCA plot and phylogenetics tree, the BH population does not segregate into a discrete cluster from other populations. While the variation in algorithms contributes to this pattern, we posited that the underlying cause was the short introduction history from China (around 50 years), the extended generational interval with 4–5 years in natural environments, and the absence of intense anthropogenic or natural selective pressures in the BH population, which lead to the low genetic differentiation between the BH population and most domestic counterparts.

Generally, the clustering pattern observed here did not correspond to geographical distribution, as observed in silver carp (*Hypophthalmichthys molitrix*) [60] and grenadier anchovy (*Coilia nasus*) [61]. Typically, frequent genetic exchange reduces the level of genetic differentiation, whereas geographic isolation, mutation, genetic drift, or artificial selection accelerate the genetic differentiation process among populations [62]. The widespread genetic similarity observed among most populations in our study was similar to findings by Zhu et al. [12] and Fu et al. [59], who noted a similar situation among *H. nobilis* populations in the middle and upper reaches of the Yangtze River using microsatellite markers and mitochondrial DNA, respectively. They attributed the similarity to similar ecological environments and anthropogenic factors. Considering the influence of anthropogenic activities and the possibility of gene flow among the sampling sites, we speculated that the intensive restocking and stock enhancement activities using mixed germplasm along the Yangtze River, followed by subsequent random mating in the natural spawning grounds, might explain the high genetic similarity observed. However, an unexpected result was that the WZ2 exhibited high genetic similarity to the YZ and YZYZ despite being over 1000 km apart, while these three populations exhibited high genetic differentiation from other populations. Previous literature showed that the natural spawning grounds of the four major Chinese carps were mainly distributed in the upper and middle reaches of the Yangtze River, whereas WZ2 was in the inundated area of the Gerges Reservoir, where changes in hydrological conditions have led to the disappearance of the original spawning grounds [63]. Thus, we tentatively speculated that the fingerlings released in WZ2 and YZ might come from the Yangzhou hatchery (YZYZ), and the absence of the spawning ground impeded the gene flow with the neighboring population, which requires further investigation. Finally, a general decline in effective population size was observed 20–30 thousand years ago in all populations during the Last Glacial Maximum (LGM), consistent with observations in a considerable number of other species, such as *Megalobrama* [44], *Mysticeti* [64], *Ectopistes migratorius* [65] and *Ailuropoda melanoleuca* [66]. The cold climate conditions and the shortage of food supplies during the LGM were likely causes of the population bottleneck. Additionally, a mild population expansion has been noticed in recent years, which might be attributed to conservation efforts.

## 5. Conclusions

In summary, we identified 1,405,783 high-quality SNPs in 157 H. nobilis from 14 populations using RAD sequencing. Population genetic analysis indicated that most populations, including the American population BH, showed low genetic differentiation. Additionally, the genetic diversity of populations generally tends to decline from upstream to downstream, with the exception of populations WZ2, YZ, and YZYZ, which were suffering heterozygote deficiency and inbreeding degradation. Overall, these findings will be instrumental in devising effective conservation and management strategies for *H. nobilis* populations in the Yangtze River basin.

## Figures and Tables

**Table 1:** Detailed information of the 14 sampled *H. nobilis* populations.

Population ID	Sample Site	Coordinates	River System	Sample Size
Jjin	Jiangjin, Chongqing, China	106.25° E, 29.27° N	upper reach of the Yangtze River	10
WZ2	Wanzhou, Chongqing, China	108.40° E, 30.80° N		10
ZX2	Zhongxian, Chongqing, China	108.08° E, 30.32° N		10
JZ	Jingzhou, Hubei, China	112.26° E, 30.30° N	middle reach of the Yangtze River	10
CLJ	Chenlinji, Hunan, China	113.16° E, 29.46° N		10
XZ	Xingzhou, Hubei, China	114.70° E, 30.59° N		10
JHK	Jiehekou, Hunan, China	113.06° E, 29.36° N		10
XZX	Xinzixian, Jiangxi, China	116.10° E, 29.49° N		10
JJ	Jiujiang, Jiangxi, China	115.94° E, 29.73° N		10
YZ	Yangzhou, Jiangsu, China	119.45° E, 32.27° N	lower reach of the Yangtze River	10
YZYZ	Yangzhou hatchery, Jiangsu, China	119.39° E, 32.23° N		10
CH	Chaohu lake, Anhui, China	117.50° E, 31.44° N		10
TH	Taihu lake, Jiangsu, China	120.04° E, 31.41° N		10
BH	the Marseilles Reach of the Illinois River	−88.74° W, 41.33° N	Mississippi River	27

**Table 2:** Genetic diversity measurement of the 14 sampled *H. nobilis* populations.

Population	*p*	*Pi*	*HE*	*HO*	*Fis*	HWE
Jjin	0.8230	0.0002	0.2567	0.2493	0.0158	6037
ZX2	0.8109	0.0010	0.2940	0.0656	0.7850	697,267
WZ2	0.7613	0.0011	0.3448	0.0897	0.7517	836,874
JZ	0.8080	0.0003	0.2732	0.2852	−0.0500	4663
CLJ	0.8040	0.0002	0.2783	0.2902	−0.0426	4688
JHK	0.8384	0.0003	0.2355	0.2515	−0.0748	4692
XZ	0.8459	0.0003	0.2252	0.2361	−0.0634	16,400
XZX	0.7485	0.0003	0.3398	0.4248	−0.2561	36,276
JJ	0.7951	0.0002	0.2847	0.3653	−0.2905	8307
CH	0.8599	0.0003	0.2116	0.2150	−0.0506	5721
YZYZ	0.7068	0.0013	0.3952	0.0811	0.8068	886,705
YZ	0.7023	0.0013	0.3997	0.0796	0.8131	919,153
TH	0.8478	0.0007	0.2439	0.0706	0.7357	228,704
BH	0.8044	0.0002	0.2818	0.2737	0.0837	25,947

Note: *p* indicates the frequency of the most frequent allele at each locus in this population; *Pi* indicates nucleotide diversity; *HE* indicates expected heterozygosity; *HO* indicates observed heterozygosity; *Fis* indicates the inbreeding coefficient; HWE indicates the number of loci found to be significantly out of Hardy-Weinberg equilibrium (*p* < 0.05).

**Table 3:** Results of the nonparametric MANOVA of the 14 sampled *H. nobilis* populations.

Source of Variation	d.f.	Sum of Squares	MeanSqs	F.Model	r^2^	Pr (>F)
Among populations	13	1187.268	91.328	16.351	59.78%	0.001 ***
Within populations	143	798.712	5.585		40.22%	
Total	156	1985.981			100.00%	

Note: *** indicates extremely significant (*p* < 0.001).

## Data Availability

The sequence data involved in this study have been deposited in NCBI with the BioProject accession number PRJNA1117638.

## Supplementary Materials

The following supporting information can be downloaded at: https://www.mdpi.com/article/10.3390/biology13100837/s1, Table S1: Statistics of sequncing data.
